# Supplementary material for: A Jasmonate-Responsive ERF Transcription Factor Regulates Steroidal Glycoalkaloid Biosynthesis Genes in Eggplant
Source: Plants (Basel). 2022 Dec 1;11(23):3336. doi: 10.3390/plants11233336 (PMC9736504; doi:10.3390/plants11233336)
Supplement: Supplementary file 1 [file plants-11-03336-s001.zip › plants-2048710-supplementary.pdf]

Figure S1

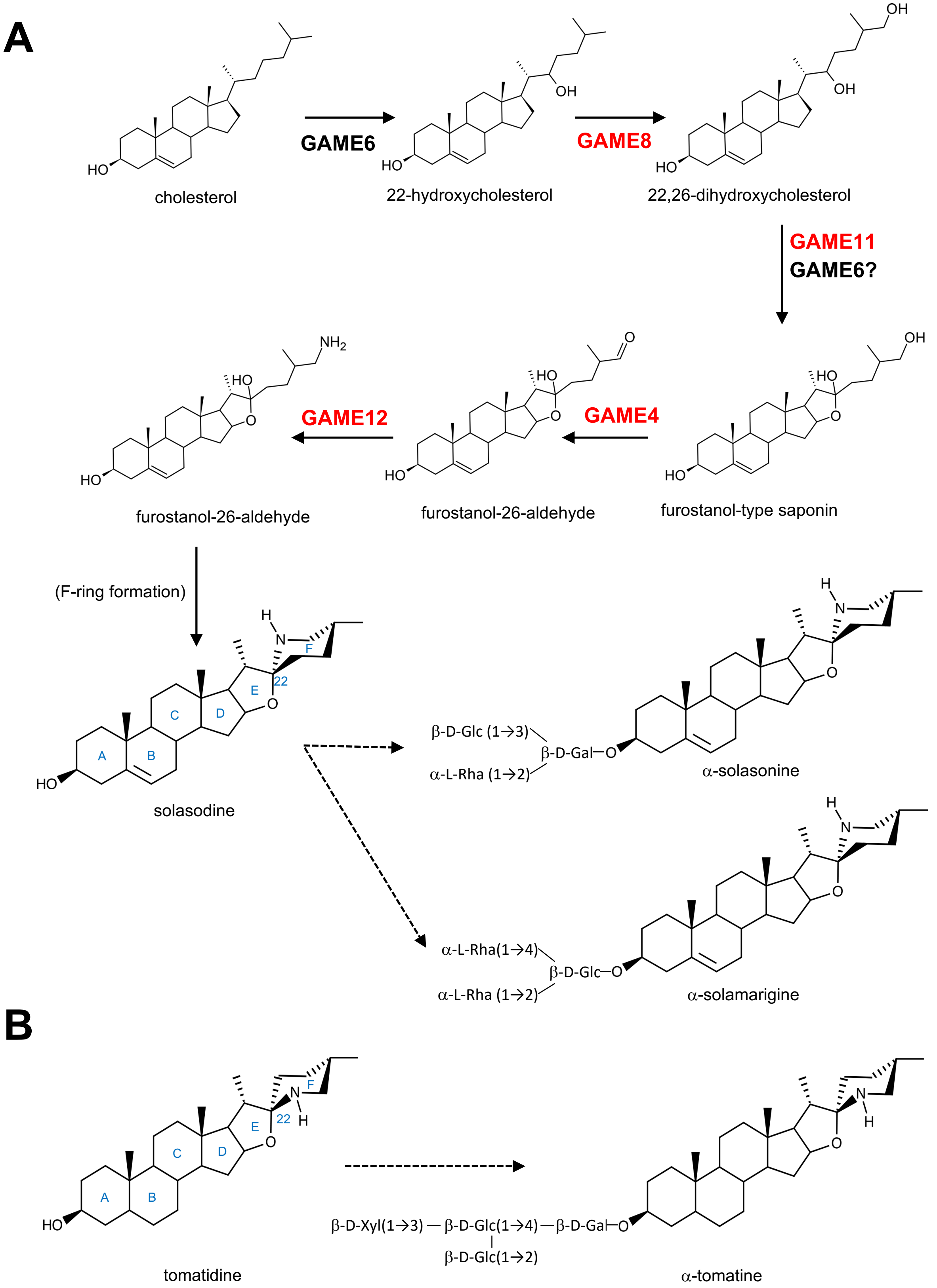

Figure S1. Part of the SGA biosynthesis pathway in eggplant (**A**) and tomato (**B**). Dashed arrows indicate steps involving multiple reactions. The rings are designated by letters (A to F) and C-22 are indicated in blue on the chemical structures of solasodine (**A**) and tomatidine (**B**). (**A**) Proposed reaction steps in the pathway converting cholesterol to the aglycone solasodine. Solasodine is further glycosylated, producing α-solasonine and α-solamargine, the major SGAs in eggplant. Names of metabolic enzymes deduced based on our criteria (see text and the legend for **Figure 1**) are shown in red; no gene encoding GAME6 (shown in black) was found. Hydroxylation at position 22 mediated by GAME6 in a step generating furostanol-type sapoin was presumed, but this has not been validated experimentally. (**B**) Tomatidine is glycosylated to α-tomatine the major SGA in tomato.

Figure S2

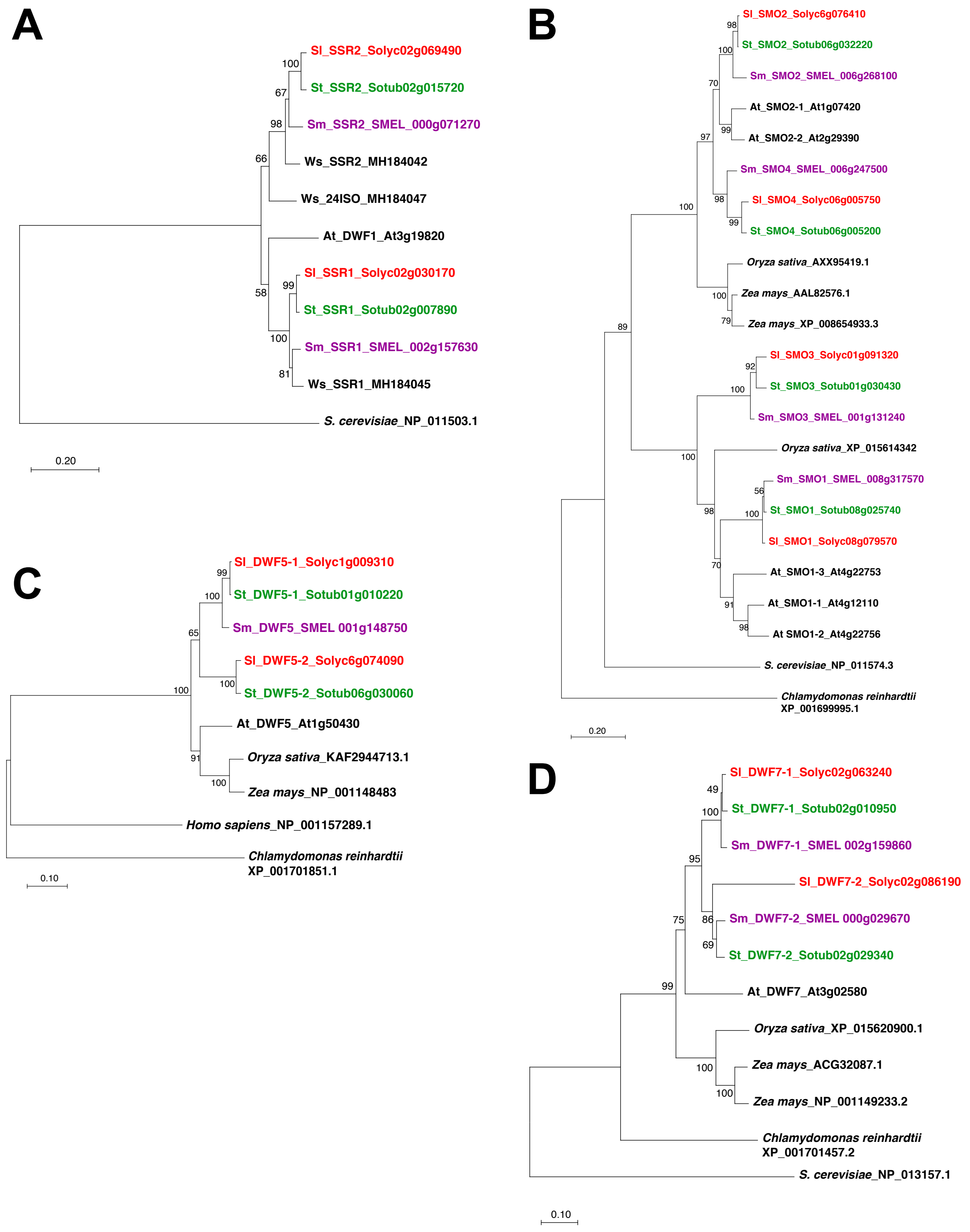

Figure S2. Phylogenetic relationship of proteins from eggplant and other species. Phylogenetic trees are shown for SSR (**A**), SMO (**B**), DWF5 (**C**), and DWF7 (**D**). Gene IDs or accession codes are indicated, and eggplant (purple), tomato (red), and potato (green) proteins are shown. The percentage support for 1,050 bootstrap values are indicated at branch nodes. The scale bar indicates the number of amino acid substitutions per site. Except for *SmDWF5* (SMEL001g137920), the eggplant and potato proteins were assigned to certain subgroups and named based on similarities to tomato counterparts.

Figure S3

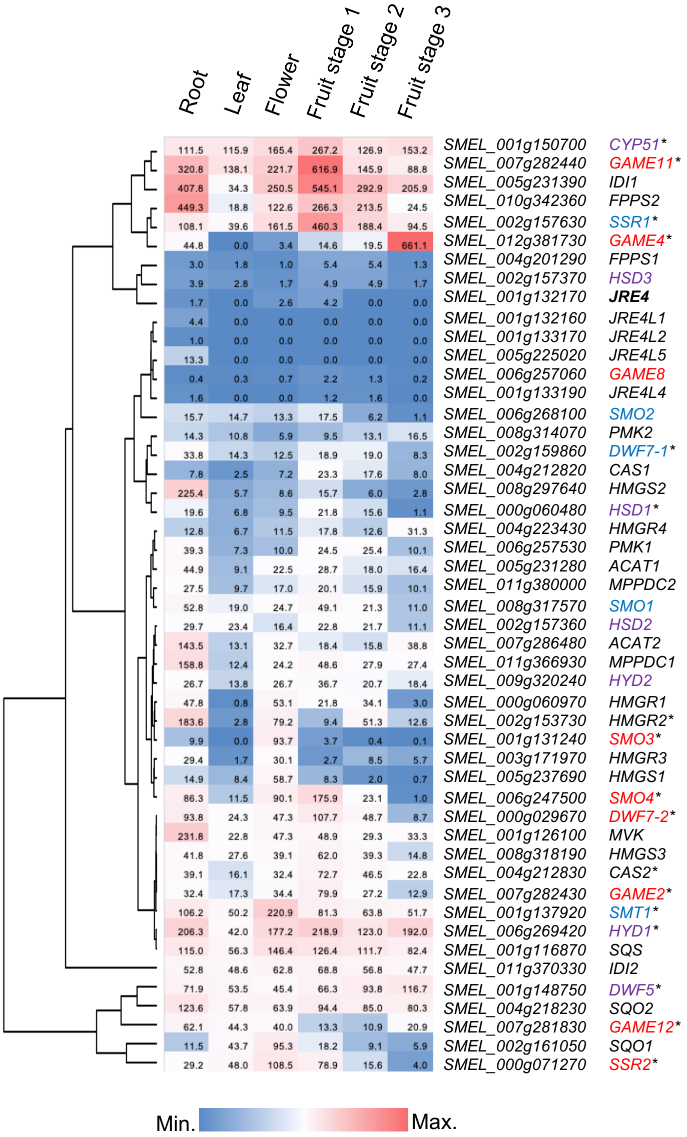

Figure S3. Heat map showing RPKM-normalized expression data [8] in eggplant tissues (roots, leaves, flowers, and fruits at stage 1 [8–10 d after pollination], fruit [skin and flesh] at stage 2 [pre-veraison, the onset of ripening], and fruit [skin and flesh] of stage 3 [physiological maturation]) of SGA and phytosterol biosynthesis genes. Hierarchical clustering was performed using the ward d2 method in R (http://www.R-project.org), and a heap map was drawn with Excel. The metabolic genes specific to SGA biosynthesis, phytosterol biosynthesis, and those shared by both are shown in red, blue, and purple, respectively. The genes examined here by RT-qPCR analyses are marked with asterisks. Primer sequences are listed in **Table S3.** Colors of shaded values represent RPKM values according to the bar.

Figure S4

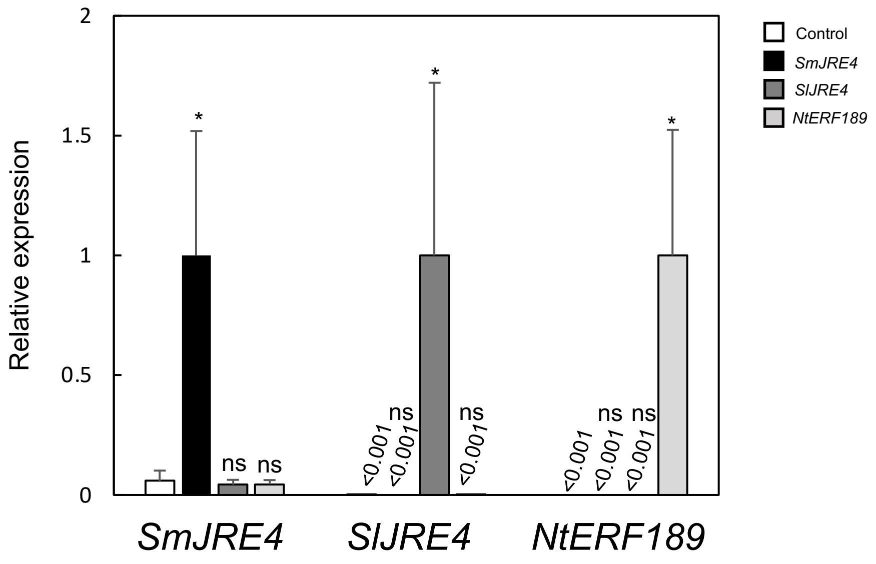

Figure S4. Transient overexpression of *SmJRE4*, *SlJRE4*, and *NtERF189* in eggplant leaves. The pGWB2-based vectors for overexpression of each ERF transcription factor gene were introduced into the leaves via *Agrobacterium*-mediated infection. At 2 d following the infection, transcript levels were analyzed by RT-qPCR. Primer sequences are listed in **Table S3**. The maximum values for each gene were set to 1. Detection of *SlJRE4* and *NtERF189* expression in the controls was considered to be nonspecific amplification. Significant differences relative to the controls were determined by Student's t-test. $^*P < 0.01$. ns; not significant.

Figure S5

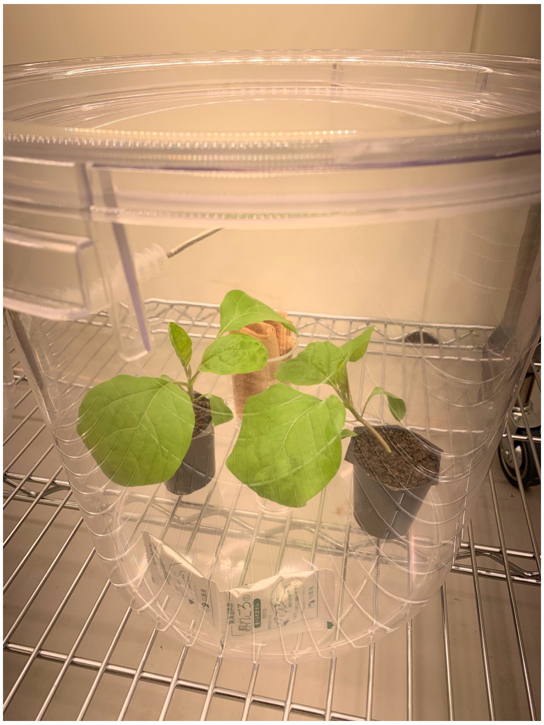

Figure S5. An eggplant treatment chamber. Eggplant plantlets exposed to gases of methyl JA (MJ) and ethylene in an air-tight bucket. A paper towel absorbed with 1.2 mL of MJ liquid and two packages of ethylene-generating agent Uregoro (Carto, Shizuoka, Japan) pierced with needles were placed in the bucket along with two individual plants.

Table S1 Tomato SGA and related phytosterol enzymatic proteins, whose sequences were used as queries for BLASTP search

name	gene ID	description
ACAT	Solyc05g017760	acetyl-CoA C-acetyltransferase
HMGS	Solyc08g080170	hydroxymethylglutaryl-CoA synthase
HMGR	Solyc02g082260	hydroxymethylglutaryl-CoA reductase
MVK	Solyc01g098840	mevalonate kinase
PMK	Solyc06g066310	phosphomevalonate kinase
MPPDC	Solyc04g009650	mevalonate diphosphate decarboxylase
IDI	Solyc04g056390	isopentenyl diphosphate isomerase
FPPS	Solyc12g015860	farnesyl diphosphate synthase
SQS	Solyc01g110290	squalene synthase
SQO	Solyc04g077440	squalene monooxygenase
CAS	Solyc04g070980	cycloartenol synthase
SSR2	Solyc02g069490	sterol side chain reductase 2
SMO3	Solyc01g091320	sterol methyl oxidase 3
SMO4	Solyc06g005750	sterol methyl oxidase 4
DWF7-2	Solyc02g086180	sterol C-5 desaturase / Dwarf 7-2
DWF5-2	Solyc06g074090	sterol reductase / Dwarf 5-2
GAME6	Solyc07g043460	CYP72 / Glycoalkaloid Metabolism 6
GAME8	Solyc06g061027	CYP72 / Glycoalkaloid Metabolism 8
GAME11	Solyc07g043420	2-oxoglutarate-dependent dioxiganase / Glycoalksaloid Metabolism 11
GAME4	Solyc12g006460	CYP88 / Glycoalkaloid Metabolism 4
GAME12	Solyc12g006470	transaminase / Glycoalkaloid Metabolism 12
GAME1	Solyc07g043490	UDP-galactosyltransferase / Glycoalkaloid Metabolism 1
GAME17	Solyc07g043480	UDP-glucosyltransferase / Glycoalkaloid Metabolism 17
GAME18	Solyc07g043500	UDP-glucosyltransferase / Glycoalkaloid Metabolism 18
GAME2	Solyc07g043410	UDP-rhamnosyltransferase / Glycoalkaloid Metabolism 2
HSD	Solyc02g032330	3β-hydroxysteroid dehydrogenase 1
CPI	Solyc12g098640	cyclopropylsterol isomerase
CYP51	Solyc01g008110	sterol C14-demethylase / CYP51
HYD2	Solyc09g009040	Δ14-sterol reductase
HYD1	Solyc06g082980	3β-hydroxysteroid-Δ8Δ7-isomerase
SMT	Solyc10g080150	sterol methyltransferase 1
SMO1	Solyc08g079570	sterol methyl oxidase 1
SMO2	Solyc06g076410	sterol methyl oxidase 2
DWF7-1	Solyc02g063240	sterol C-5 desaturase / Dwarf 7-1
DWF5-1	Solyc01g009310	sterol reductase / Dwarf 5-1
SSR1	Solyc02g030170	sterol side chain reductase 1

Table S2 Genes involved in SGA biosynthesis and related pathways in eggplant.
Genes, whose expression was analyzed by RT-qPCR, are colored in yellow. Primers used to detect them are listed in **Table S3**.

gene name	gene ID	description
ACAT1	SMEL_005g231280	acetyl-CoA C-acetyltransferase 1
ACAT2	SMEL_007g286480	acetyl-CoA C-acetyltransferase 2
HMGS1	SMEL_005g237690	hydroxymethylglutaryl-CoA synthase 1
HMGS2	SMEL_008g297640	hydroxymethylglutaryl-CoA synthase 2
HMGS3	SMEL_008g318190	hydroxymethylglutaryl-CoA synthase 3
HMGR1	SMEL_000g060970	hydroxymethylglutaryl-CoA reductase 1
HMGR2	SMEL_002g153730	hydroxymethylglutaryl-CoA reductase 2
HMGR3	SMEL_003g171970	hydroxymethylglutaryl-CoA reductase 3
HMGR4	SMEL_004g223430	hydroxymethylglutaryl-CoA reductase 4
MVK	SMEL_001g126100	mevalonate kinase
PMK1	SMEL_006g257530	phosphomevalonate kinase 1
PMK2	SMEL_008g314070	phosphomevalonate kinase 2
MPPDC1	SMEL_011g366930	mevalonate diphosphate decarboxylase 1
MPPDC2	SMEL_011g380000	mevalonate diphosphate decarboxylase 1
IDI1	SMEL_005g231390	isopentenyl diphosphate isomerase 1
IDI2	SMEL_011g370330	isopentenyl diphosphate isomerase 1
FPPS1	SMEL_004g201290	farnesyl diphosphate synthase 1
FPPS2	SMEL_010g342360	farnesyl diphosphate synthase 2
SQS	SMEL_001g116870	squalene synthase
SQO1	SMEL_002g161050	squalene monooxygenase 1
SQO2	SMEL_004g218230	squalene monooxygenase 1
CAS1	SMEL_004g212820	cycloartenol synthase 1
CAS2	SMEL_004g212830	cycloartenol synthase 1
SSR2	SMEL_000g071270	sterol side chain reductase 2
SMO3	SMEL_001g131240	sterol methyl oxidase 3
SMO4	SMEL_006g247500	sterol methyl oxidase 4
DWF7-2	SMEL_000g029670	sterol C-5 desaturase / Dwarf 7-2
GAME8	SMEL_006g257060	CYP72 / Glycoalkaloid Metabolism 8
GAME11	SMEL_007g282440	2-oxoglutarate-dependent dioxiganase / Glycoalksaloid Metabolism 11
GAME4	SMEL_012g381730	CYP88 / Glycoalkaloid Metabolism 4
GAME12	SMEL_007g281830	transaminase / Glycoalkaloid Metabolism 12
GAME2	SMEL_007g282430	UDP-rhamnosyltransferase / Glycoalkaloid Metabolism 2
HSD1	SMEL_000g060480	3β-hydroxysteroid dehydrogenase 1
HSD2	SMEL_002g157360	3β-hydroxysteroid dehydrogenase 2
HSD3	SMEL_002g157370	3β-hydroxysteroid dehydrogenase 3
CYP51	SMEL_001g150700	sterol C14-demethylase / CYP51
HYD2	SMEL_009g320240	Δ14-sterol reductase
HYD1	SMEL_006g269420	3β-hydroxysteroid-Δ8Δ7-isomerase
DWF5	SMEL_001g148750	sterol reductase / Dwarf 5
SMT1	SMEL_001g137920	sterol methyltransferase 1
SMO1	SMEL_008g317570	sterol methyl oxidase 1
SMO2	SMEL_006g268100	sterol methyl oxidase 2
DWF7-1	SMEL_002g159860	sterol C-5 desaturase / Dwarf 7-1
SSR1	SMEL_002g157630	sterol side chain reductase 1
JRE4	SMEL_001g132170	Jasmonate-responsive ERF 4
JRE4L1	SMEL_001g132160	JRE4-like 1
JRE4L2	SMEL_001g133170	JRE4-like 2
JRE4L3	SMEL_001g133180	JRE4-like 3
JRE4L4	SMEL_001g133190	JRE4-like 4
JRE4L5	SMEL_005g225020	JRE4-like 5

Table S3 Oligonucleotide primers used in RT-qPCR analysis

gene name	gene ID	forward/reverse (F/R)	sequence (5' to 3')
Sm HMGR2	SMEL_002g153730	F	ATGGACGTTCGCCGGAGATCTG
		R	CAGAGGCATCAATGAGAAGGGTGTC
Sm CAS2	SMEL_004g212830	F	AATGTTTTGTTTTCCTTACAGAATAGTGG
		R	TTGCAGCTGAGGAACACTCTACATACGG
Sm SSR2	SMEL_000g071270	F	AGCAATATGTAAAACTTACCTACAAACCTG
		R	CTACCATCTCTGGAACTTTAGAAGAATTGTC
Sm SMO3	SMEL_001g131240	F	TTGCCATTTGGGAGCATTGAAGAG
		R	GGTGTTGTGACAATATAGATAGAAATCTG
Sm SMO4	SMEL_006g247500	F	ATGGCCTCCATGATCGAATCTGC
		R	GAGTCCAGACAAGAAGAAGGCACTC
Sm DWF7-2	SMEL_000g029670	F	AACAAGCAAAACACACTTTCCCCGTTTGCTG
		R	GGAATATGAGCCCTATGTGTGTAGTGAAATGC
Sm GAME11	SMEL_007g282440	F	GACCTCCTCTCCAACTATTCAAGC
		R	TAGCTTTTCCCAAATCAATTACTGG
Sm GAME4	SMEL_012g381730	F	AAGCCAAGCATTATTATCACAAAGCCAG
		R	CTTATCTTCTTCGATTGATGTCGATAGTCC
Sm GAME12	SMEL_007g281830	F	CAAAACCTTCTTTGGATCTTGCAAAGGAGC
		R	GCATTGTTGTAATACCAGACCAGCTTCACCTGG
Sm GAME2	SMEL_007g282430	F	ATGGCAACGGAAGTGAAGGAAC
		R	GCCATGGAGGGCGAAGAGTCTAGC
Sm HSD	SMEL_002g157360	F	TTCATAGCTTCTGGCTTCACC
		R	CCATTATTCTCTGAGGAAGTCTCC
Sm CYP51	SMEL_001g150700	F	ATGGAGTTAGGTGACAACAAGATTCTG
		R	CCATGATTTGATCACTGGAGGCAAACG
Sm HYD2	SMEL_009g320240	F	CTCCATTGACACTGCATATTAGGC
		R	GAAGGAGTGAGAGAAATGAG
Sm HYD1	SMEL_006g269420	F	ATGACAATTCAGGGAGAAGCTCAC
		R	CAGGAAGGATGAGATGCCATACACAC
Sm DWF5	SMEL_001g148750	F	ATGGCAGAGACCAAGTTGGTACAC
		R	CATCAGCGTGTACATTTGTATACC
Sm SMT1	SMEL_001g137920	F	GCACGGCGCTTTTGATCTGGC
		R	CTTCTTCTTCACCTCCATAATAACC
Sm DWF7-1	SMEL_002g159860	F	ACGACTACTTGAATTTGTTTGTCG
		R	GCGGAGCCATCCTTGAAACCAATGAG
Sm SSR1	SMEL_002g157630	F	ATGCTATACCATGGTCTCAAGGGACTCTG
		R	AAGCCTGTGCAATCTCTTTCAGATTACC
Sm JRE4	SMEL_001g132170	F	CGATTTTTCTCCAAGTATTTCGTCG
		R	CTTTGTTTCCTCCGGGGGCTCACATCC
Sm CYP	SMEL_001g116150	F	ATCCTTGTCCATGGCTAATGC
		R	ATGCCCTCAACAACTTGTCC
Sl JRE4	Solyc01g090340	F	CGATTTTTTCGAAACTCTTTCC
		R	TGTTTCCTCCGGTGTTACGG
Nt ERF189	AB827951.1	F	GCAGCTTCGACTGCAGCTTCCTC
		R	CTCCTCGGACTCGGAGCACTTC
